# Growth characteristics and morphology of *Paramoeba perurans* from Atlantic salmon *Salmo salar* L. and ballan wrasse *Labrus bergylta* in Norway

**DOI:** 10.1186/s13071-023-05715-2

**Published:** 2023-03-23

**Authors:** Steffen Blindheim, Linda Andersen, Christiane Trösse, Egil Karlsbakk, Are Nylund

**Affiliations:** 1 Department of Biological Sciences, University of Bergen, 7803, 5020 Bergen, Norway; 2 The Industrial and Aquatic Laboratory, Thormøhlensgate 55, 5006 Bergen, Norway

**Keywords:** Amoebic gill disease, AGD, Growth curve, Amoebae, Clonal culture, Salinity, Temperature

## Abstract

**Background:**

*Paramoeba perurans* is the causative agent of amoebic gill disease (AGD) in Atlantic salmon *Salmo salar* L. and many other farmed marine fish species worldwide. The first cases of AGD in Norway were reported in 2006, and it has subsequently become established as a significant gill disease that affects the country’s salmonid aquaculture industry. Despite several decades of research on AGD, there is still a lack of knowledge of the biology of *P. perurans* and its interactions with its hosts and the environment.

**Methods:**

The growth and morphology of 10 clonal isolates of *P. perurans* were studied. The isolates were from farmed Atlantic salmon and ballan wrasse that had been obtained from different sites along the Norwegian coast between 2013 and 2015. The morphology and population growth patterns of these clonal amoeba isolates were examined in vitro using light microscopy and real-time reverse transcription polymerase chain reaction under a range of temperatures (4, 12, 15 and 21 °C) and salinities (20, 25, 30 and 34 ‰).

**Results:**

We found distinct morphological differences between both locomotive and floating forms of the amoeba isolates. The locomotive amoebae of the clonal isolates varied in size (area) from 453 µm^2^ to 802 µm^2^. There were differences in the growth patterns of the clonal amoeba isolates under similar conditions, and in their responses to variations in temperature and salinity. While most of the isolates grew well at salinities of 25–34 ‰, a significant reduction in growth was seen at 20 ‰. Most of the amoeba isolates grew well at 12 °C and 15 °C. At 4 °C, amoebae grew slower and, in contrast to the other temperatures, no extended pseudopodia could be seen in their floating form. The isolates seemed to reach a plateau phase faster at 21 °C, with a higher number of smaller, rounded amoebae.

**Conclusions:**

The differences observed here between clonal isolates of *P. perurans* should be further examined in experimental in vivo challenge studies, as they may be of relevance to the virulence and proliferation potential of this amoeba on gills. Potential differences in virulence within *P. perurans* could have implications for management strategies for AGD.

**Graphical Abstract:**

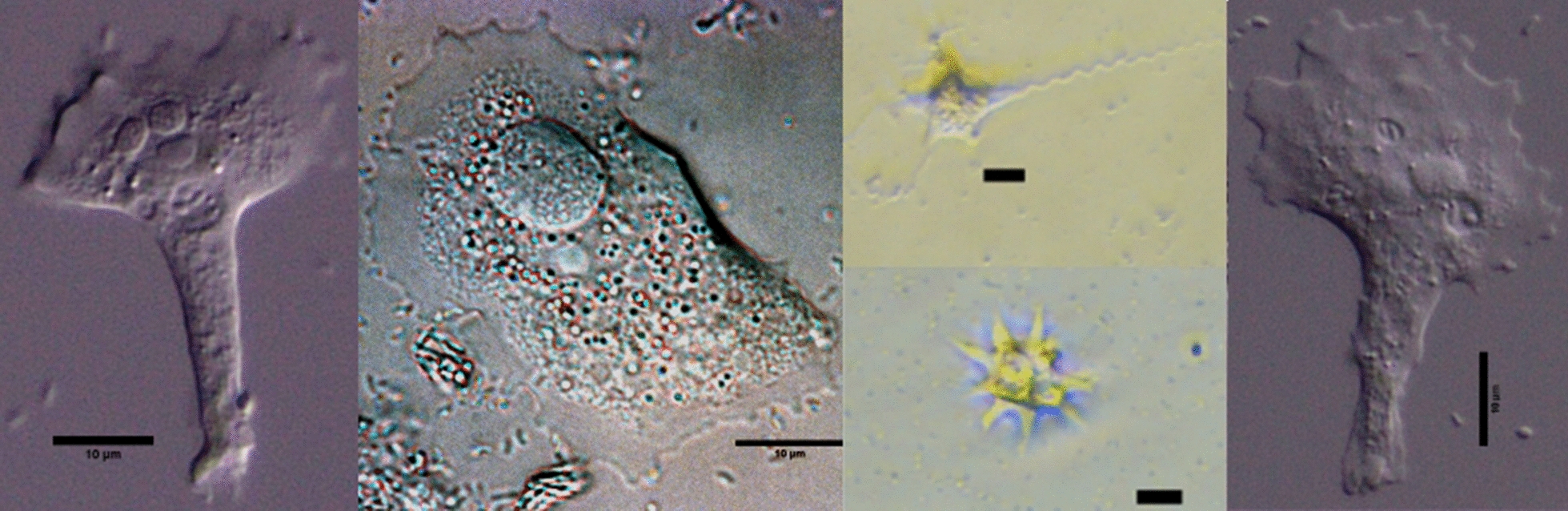

**Supplementary Information:**

The online version contains supplementary material available at 10.1186/s13071-023-05715-2.

## Background

Amoebic gill disease (AGD) in seawater-farmed Atlantic salmon *Salmo salar* is caused by the amoeba *Paramoeba perurans* (syn. *Neoparamoeba perurans* [1]) [2, 3]. AGD has been reported from salmonids and unrelated fish species such as turbot *Scophthalmus maximus*, ayu *Plecoglossus altivelis*, ballan wrasse *Labrus bergylta*, and lumpfish *Cyclopterus lumpus* [4–8]. It has been a serious problem for salmon aquaculture in Tasmania for several decades [9, 10], and has more recently become a major concern in other salmon-producing countries such as Ireland, Scotland, and Chile [9]. In Norway, AGD has been a recurring problem since 2013 [5]. Although the amoeba is now widespread in Norwegian aquaculture, the severity of AGD outbreaks vary. It has not been established if this is mostly related to variations in environmental conditions or host status, or if differences in virulence between amoeba isolates influence the severity and outcome of an infection.

The gross pathology of AGD is characterized by pale mucoid patches on the gills [11], while histopathological gill lesions include areas with epithelial hyperplasia and fusion of secondary lamellae [10]. The disease has been well described, but there are few publications on *Paramoeba perurans*’s biology and interactions with the environment [12]. This is partly because *P. perurans* was not identified until 2007 [3], and before this other paramoebae, specifically *Paramoeba pemaquidensis*, were assumed to be the causative agents of AGD [8]. Furthermore, methods for isolating and maintaining *P. perurans* in culture were not published until 2012 [2]. An increasing number of studies looking into the characteristics, biology and virulence traits of amoebae have been published [12–24] since *P. perurans* was shown to be the aetiological agent of AGD. Morphological characteristics identified through light and electron microscopy have been used for many years to identify and group amoebae [25]. More recently, molecular methods have become increasingly important for their identification and classification. In particular, 18S ribosomal RNA (rRNA) gene sequences have been used to resolve phylogenetic relationships of amoebae [26].

Amoebae of the genus *Paramoeba* are ubiquitous in marine environments, and some of them are pathogenic to marine animals such as fish (*Paramoeba perurans*), lobsters (*Paramoeba perniciosa*), and sea urchins (*Paramoeba invadens*). *Paramoeba* spp*.* are small, lobose amoebae with dactylopodiate pseudopodia when in their locomotive or attached form [25, 27]. The free-living species may show cell surface structures (microscales, hemispheres, tubule-like structures), while animal symbionts such as *Paramoeba invadens*, *Paramoeba branchiphila* and *Paramoeba perurans* show a smoother glycocalyx [28, 29]. One of the main identifying characteristics of members of the genus *Paramoeba*, which is shared with the closely related genus *Janickina*, is the presence of one or more parasomes. This juxtanuclear organelle has been shown to be a pro-kinetoplastid endosymbiont similar to *Perkinsela amoebae*, a symbiont in *Janickina pigmentifera*, and is therefore often referred to as a *Perkinsela amoeba*-like organism (PLO) [24, 30, 31]. The relationships between PLOs and species of these genera are believed to be obligatory and mutualistic, as most of the latter harbour at least one parasome (between one and six have been reported) [32–34]. The parasomes are believed to be vertically transmitted from parent to daughter cell [24]. In addition, *P. perurans* may harbour endosymbiotic bacteria such as ‘*Candidatus* Syngnamydia salmonis’ [21] or *Vibrio* species [35]. The role of these endosymbionts, if any, is currently not known, but several other amoebae such as *Acanthamoeba castellanii* and *Hartmanella vermiformis* are known to harbour *Chlamydia*-like bacteria (see e.g. Horn et al. [36]). In culture, *Paramoeba* spp. can be seen as adherent, flat locomotive forms, or star-shaped floating forms with long, slender pseudopods [23, 25, 28]. When *P. perurans* cultures age, an increasing number of small, rounded floating forms of the amoeba may occur, which are known as pseudocysts [19]. Pseudocysts are viable, and though not true cysts, these forms may enable the amoeba to survive under unfavourable environmental conditions.

Important risk factors for outbreaks of AGD are high salinity (> 32 ‰) and temperature (> 12 °C), although AGD has been reported at lower temperatures and salinities [8, 37]. In non-clonal cultures of *P. perurans* obtained from salmon on the west coast of Scotland, population growth was arrested at temperatures below 8 °C and above 18 °C [12]. Growth was also arrested below 20–25 ‰, while the conditions for optimal growth were reported to be 15 °C and 35 ‰, with amoeba numbers doubling every 14 h [12].

The extent of variation in amoeba strains and its significance to virulence during an AGD infection of Atlantic salmon are not known. Hence, we examined the morphological characteristics of 10 *P. perurans* clonal cultures derived from salmon and ballan wrasse from different geographical areas of Norway, together with growth curve patterns and survival at a range of temperatures (4, 12, 15 and 21 °C) and salinities (20, 25, 30 and 34 ‰).

## Methods

### Primary isolation and culturing of* P. perurans*

Amoebae were isolated from the gills of individual, killed fish showing signs of AGD that had been sent to either the Fish Disease Research Group at the University of Bergen (Bergen, Norway) or to the Industrial and Aquatic Laboratory (ILAB; Bergen, Norway) from commercial fish farms between 2013 and 2015 (Table 1). Most of the isolates were isolated from fish in the autumn months (August–November), when the incidence of AGD was high along the Norwegian coast. Isolate 8, however, was isolated from an AGD case in February, and isolate 3 was isolated in January from a tank experiment with net-pen-reared Atlantic salmon, which had been brought in from a fjord outside Bergen to ILAB in 2013, in which *P. perurans* was an incidental finding when the gills were examined [38]. The approval identifiers for the animal experiments conducted at the ILAB are given in the “Ethics approval and consent to participate” section. The isolates were obtained by scraping mucus and gill tissue with amoebae onto malt yeast agar (MYA) plates and adding 10–15 mL of autoclaved seawater [2]. Isolate 4 was isolated from the gills of a ballan wrasse held in a wrasse-only land-based facility [18]. Plates were washed with autoclaved seawater the next day to remove debris and excess bacteria. All plates were incubated at 15 °C in a Sanyo MIR-554 incubator (Sanyo Electric, Osaka, Japan). Upon confirmation of amoeba growth (the amoebae were observed using an inverted microscope) a small piece of agar with amoebae was transferred to a new MYA plate and a layer of autoclaved seawater was added. For subsequent passages, the amoebae were either transferred to cell culture flasks (Nunclon) and grown in malt yeast broth (MYB) [5, 21], or transferred to new MYA plates, as described above.

**Table 1:** An overview of the *Paramoeba perurans* isolates included in the study

Isolate	UoB identifier	Fish species	County	Year	Months since first isolation			
					Morphology	Growth at 15 °C	Temperature	Salinity
1	H01/13Pp B2	*Salmo salar*	Hordaland^a^	2013	27	17	24	21
2	H02/13Pp C2	*Salmo salar*	Hordaland	2013	59	18	25	22
3	H03/14Pp B10	*Salmo salar*	Hordaland	2014	25	22	22	20
4	H04/14Pp F3	*Labrus bergylta*	Hordaland	2014	18	8	15	18
5	MR06/14Pp E4	*Salmo salar*	Møre and Romsdal	2014	47	12	12	10
6	SF07/14Pp B2	*Salmo salar*	Sogn and Fjordane^a^	2014	48	7	14	11
7	SF08/14Pp E1	*Salmo salar*	Sogn and Fjordane	2014	17	14	14	11
8	SF11/15Pp C4	*Salmo salar*	Sogn and Fjordane	2015	13	10	10	13
9	R18/15Pp D7	*Salmo salar*	Rogaland	2015	6	3	3	6
10	ST19/15Pp K2	*Salmo salar*	Trøndelag	2015	4	1	1	5

The University of Bergen (*UoB*) identifier is based on county, culture number, year, species and cell culture tray well of the clone. The isolates are numbered according to the time of isolation. The age of the cultures at the start of each experiment is given in months

^a^Hordaland and Sogn and Fjordane are now collectively known as Vestland county

### Clonal cultures

Clonal strains of each isolate were established by using two different methods [18, 21]. Briefly, method 1 consisted of transferring single floating amoebae from MYA plates or cell culture flasks into individual wells in 24-well cell culture plates. Method 2 involved seeding individual amoebae that had been diluted with MYB from cell culture flasks into wells of a 96-well cell culture plate. For the latter approach, the dilution of the original amoebae polyculture was such that only a few of the 96 wells receiving MYB would contain amoebae. For both methods, microscopy was used to ensure that only one amoeba was present in the wells. The clonal *P. perurans* isolates (isolate 1–10) used in this study are listed in Table 1.

### DNA extraction, polymerase chain reaction and sequencing

To confirm the identity of the amoeba clones, partial sequencing of the 18S rRNA gene was conducted on DNA extracted from all clones of *P. perurans* used in this study. Both amoebae attached to the substrate and floating amoebae were concentrated by centrifugation at 1430 × *g* for 15 min and resuspended in 1 mL seawater. The amoebae were then centrifuged for 5 min at 21,000 × *g* and the resulting pellets were resuspended in 200 µl phosphate buffered saline for DNA extraction using the DNeasy Blood & Tissue kit (Qiagen) as recommended by the manufacturer. DNA was stored at −20 °C before use.

Polymerase chain reaction (PCR) and sequencing were performed as described in [18], except that NP-F2 (forward, 5′-GCATGGGATAATGGAACAGG-3′) and NP-R10 (reverse, 5′-AAGTTTACCCCATCCTTTCG-3′) primers and an annealing temperature of 57 °C were used. The resulting sequences were trimmed to 736 base pairs and compared to the rRNA gene from *P. perurans* (EF216904 [3]).

### RNA extraction and real-time reverse transcription PCR

Real-time reverse transcription PCR (RT-PCR) was used for the relative quantification of amoeba RNA from the different experiments to establish growth curves and compare development. RNA from each in vitro experiment was extracted as described in Gunnarsson et al. [39]. For further real-time RT-PCR analyses the AgPath-ID one-step RT-PCR kit (Applied Biosystems) was used together with the 18S rRNA Pperu assay [21]. As no endogenous control was available, the obtained Ct values are presented in this paper as ‘load’, i.e. the Ct value is subtracted from the number of cycles run (45). In addition, all the isolates were checked for the presence of ‘*Candidatus* Syngnamydia salmonis’ using the SCh assay [40] since this bacterium has been found to live intracellularly in *P. perurans* [21].

### Morphological evaluation and size measurements

*Paramoeba perurans* grown in cell culture flasks with MYB were harvested by cell scraping and centrifugation at 1000 × *g* for 15 min. The supernatant was discarded, and the resulting pellets were resuspended in MYB to obtain a concentration of amoebae suitable for microscopy. A 10-µl amoeba suspension was used to make each hanging drop preparation. The amoebae were allowed to attach and establish locomotion for 1 h before the preparations were examined by Nomarski differential interference contrast microscopy on a Zeiss Axioskop 2 Plus microscope (isolates 1, 3, 4, 7–10) and photographed using a Nikon digital sight DS-5 M camera. For isolates 2, 5 and 6, an Olympus BX43 microscope with an SC50 camera was used. The amoebae were measured on captured photos using the software ImageJ 1.50B (http://imagej.nih.gov/ij). Images of 40–60 individual amoebae from each isolate were studied to obtain at least 30 measurements per isolate. When the outlines of nuclei or parasomes were obscured by the granular endoplasm (unclear), they were not measured, hence fewer measurements were obtained for these organelles. The measurements of the amoebae were based on locomotive (actively moving) amoebae in hanging drop preparations. ‘Length’ was measured from the distal part of the leading hyaline cytoplasmic edge (‘anterior’) to the cytoplasmic edge of the trailing end with granular endoplasm and/or uroid(s) (‘posterior’). The ‘width’ was then measured perpendicular to the length.

### Amoeba population growth curves at 15 °C

Cell culture plates with 24 wells were used for the experiments conducted to determine *P. perurans* population growth. Two experiments were conducted with similar set-ups, but at different times and with different starting concentration of amoebae and numbers of sampling points. In experiment 1, the wells for isolates 1, 2, 4 and 6 were inoculated with 600–1900 amoebae (determined by using a CASY cell counter [5]) in 500 µl MYB, while in experiment 2, the wells for isolates 3, 5, 7–10 were inoculated with approximately 500 amoebae (determined by using a Bürker cell counting chamber) in 500 µl MYB. For both experiments, the amoebae were incubated in the dark and kept undisturbed at 15 °C in a Sanyo MIR-554 (Sanyo Electric) incubator until sampling. Samples were collected after 2 hours and at 1, 2, 3, 4, 8, 11 (for experiment 1 only) and 14 days post-inoculation (dpi), by transferring the supernatant (containing floating amoebae) from each well to individual 1.5-ml Eppendorf tubes. Then, 1 mL Isol RNA-lysis reagent (5 PRIME, Hamburg, Germany) was added to each tube, and 1 mL Isol RNA-lysis reagent (5 PRIME) was added to each well. The well contents were mixed and then transferred to separate 1.5-ml Eppendorf tubes (this latter fraction contained amoebae attached to the bottom of the wells). The samples were stored at −40 °C until RNA extraction.

### Temperature tolerance

In addition to the growth characteristics experiment conducted at 15 °C, the growth of all 10 *P. perurans* isolates was assessed at three additional temperatures in an identical set-up to experiment 2. For each temperature (4, 12 and 21 °C) and sampling point (3, 8 and 14 dpi), approximately 500 amoebae (determined by using a Bürker cell counting chamber) were inoculated into three replicate wells containing 500 µl MYB. A fridge was used for the 4 °C incubation, a Minitron incubator (Infors) for the 12 °C incubation; the 21 °C incubation was at room temperature. Floating amoebae were sampled by transferring the supernatant to individual 1.5-ml Eppendorf tubes, centrifuging the supernatant at 16,200 × *g* for 5 min, then removing the supernatant and freezing the pellet at − 40 °C. Attached amoebae were collected by adding 1 ml Isol RNA-lysis reagent (5 PRIME) to the well, mixing and transferring the liquid to a separate 1.5-ml Eppendorf tube and freezing at − 40 °C until the RNA was extracted as described above.

In 2021, an experiment was conducted to test recovery after an extended cold period. One isolate, isolate 2, that had been held at ILAB for 9 years and shown to be virulent during this period, was split into three 75-cm^2^ cell culture flasks and held at 15 °C in a series KB8182 incubator (Termaks). After 3 days, the three bottles were transferred to 4 °C. After an extended period (37 days and 57 days), cells were scraped from about one third of the surface area per cell culture flask, and together with one third of the supernatant transferred into new cell culture flasks. The cultures in the new flasks were supplied with MYB, incubated under standard conditions, and after 8 and 20 days checked for viability, which was assessed as the presence of attached amoebae using an inverted microscope (DM IRBE; Leica). The amoebae were counted and the numbers compared to those of amoebae seen during normal maintenance passages.

### Salinity tolerance

The growth of *P. perurans* clones was assessed in two similar experiments at four different salinities: 20, 25, 30 and 34 ‰. The different salinities were achieved with pre-mixed MYB and distilled H_2_O and measured using MultiLine 3420 Portable Digital Multiparameter apparatus (WTW). Five hundred microlitres of solution at each salinity was added to six wells in a 24-well cell culture plate, with one plate per isolate. The amoebae were harvested from cell culture flasks by centrifugation as described earlier, and approximately 200 amoebae were added to each well. The cell culture plates were incubated at 16 °C in a Sanyo MIR-554 (Sanyo Electric) incubator and at 6 (experiment 2, isolates 8–10) or 7 (experiment 1, isolates 1–7) and 17 dpi (all isolates), two wells of each salinity for all isolates were harvested by transferring the supernatant to a 1.5-mL Eppendorf tube. Two hundred and fifty microlitres of MYB was then added to each well and mixed to loosen attached amoebae into suspension. The 250 µl of MYB containing previously adherent amoebae was transferred to the same centrifuge tube as the floating amoebae and centrifuged at 12,000 × *g* for 5 min at 4 °C. All the supernatant but 50 µl was discarded, and the tubes were frozen at − 40 °C. RNA was extracted as previously described after the addition of 1 ml Isol RNA-lysis reagent (5 PRIME). At 20 dpi, the amoebae in the two remaining wells at each salinity were detached, pooled, and transferred to a new well (one per salinity) in a 6-well cell culture plate. Five millilitres of 34 ‰ MYB was added to each well and the plates were incubated at 16 °C in a Sanyo MIR-554 (Sanyo Electric) incubator for 11 days to assess amoebae viability after prolonged exposure to variable salinities.

### AGD-challenge study: 25 and 34 ‰

An AGD-challenge study was conducted in 2014 where 30 salmon (individual weight ~ 90 g, range 69.9–100.9 g at first sampling) challenged with amoebae (isolate 2, 1000 amoebae per litre, 50-L volume) for 1 h at 34 ‰ were transferred directly after the challenge to either 25 or 34 ‰, with 15 salmon in each tank (500-L tanks). Isolate 2 was chosen due to its virulence for salmon in other challenge studies [5]; the inoculum was prepared as described in Haugland et al. [5]. Five fish were sampled from each tank at 15 and 27 days post-challenge (dpc). The gill score was assessed using the gill arch with the most severe lesions, as described in Haugland et al. [5], and gill samples were collected for real-time RT-PCR analysis and re-isolation of amoebae on MYA. The gill samples were analysed using the NeoUni [13] and EF1A [41] assays at PHARMAQ Analytiq (Bergen, Norway). Presence of surviving amoebae was examined as growth on MYA plates and examined under an inverted microscope (Leica DM IRBE; Leica). The animal study was conducted under the animal approval identifier 6926.

### Statistics

Dimensions of the amoebae of the different isolates were compared using non-parametric Kruskal–Wallis tests followed by post hoc multiple comparisons. Amoebae growth in cultures was analysed on load (45-Ct) data using ANOVA and Newman-Keuls post hoc test. Heteroscedasticity was examined with Levene’s test;* x*^*n*^ transformations were used when necessary. Correlations were examined using Spearman’s rank-order correlation coefficients (*r*_s_). Statistical analyses were performed with the software Statistica 13.

## Results

### PCR and sequencing

All 10 clonal isolates of the amoeba were confirmed to be *P. perurans* by sequencing, and all of these clones, except H02/13Pp C2, were positive for *‘Candidatus* Syngnamydia salmonis’ by real-time RT-PCR. Alignment of the partial rRNA gene from the isolates with EF216904 is shown in Additional file 1: Alignment A1.

### Morphology of locomotive amoebae

**Fig. 1 a–f:**
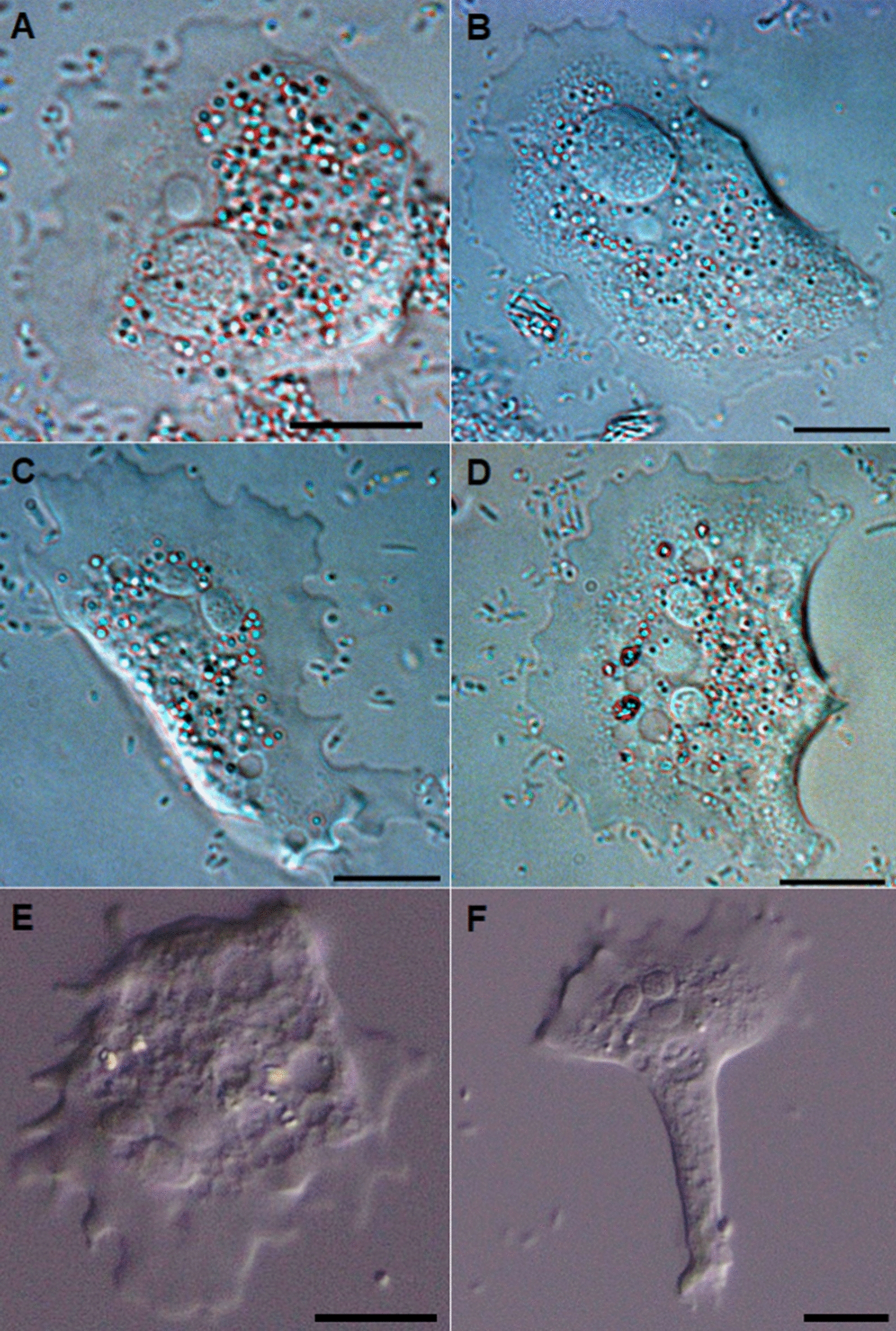
Morphology of locomotive *Paramoeba perurans* in hanging drop preparations. *Paramoeba perurans* with* Perkinsela*-like organisms (parasomes) of greatly increased size (**a**, **b**). *Paramoeba perurans* with two parasomes (**c**–**f**). Scale bars represent 10 µm. **a** Isolate 1; **b** isolate 7; **c** isolate 8; **d** isolate 3; **e**, **f** isolate 2

**Fig. 2 a–g:**
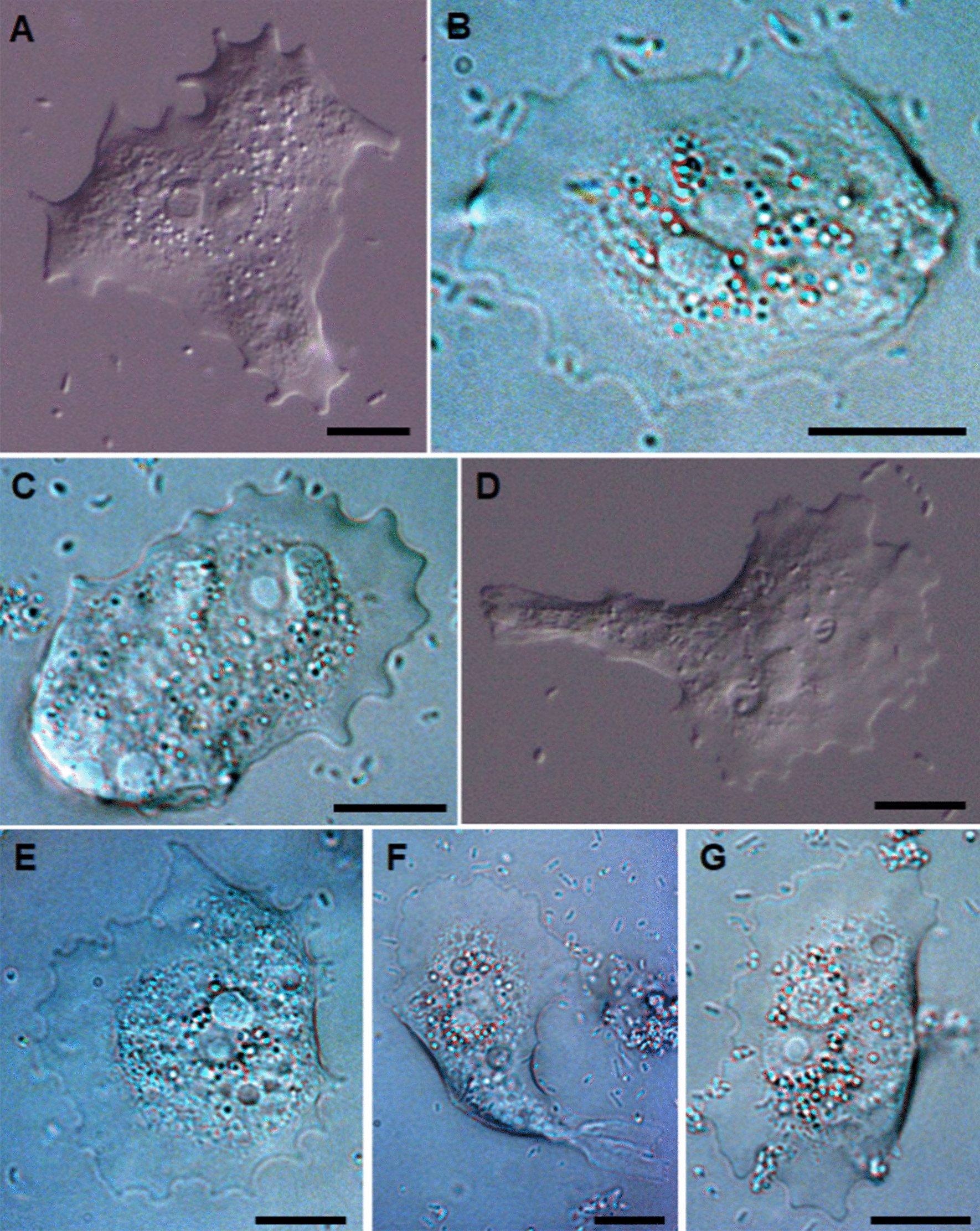
Different morphologies of locomotive *Paramoeba perurans* in hanging drop preparations. **a** Isolate 5, **b** isolate 10, **c** isolate 7, **d** isolate 2, **e** isolate 4, **f** isolate 9, **g** isolate 1. Scale bars represent 10 µm

When observed in hanging drop preparations, actively moving trophozoites of the 10 strains displayed similar morphotypes (Figs. 1, 2). They were irregular in shape, usually oval, but also had triangular to flabellate, spatulate or even bilobed shapes. Their length was normally less than their width (Table 2). In all of the isolates, the trophozoites showed an anterior and often laterally flattened hyaloplasmic rim, with 10–20 short subpseudopodia rarely projecting more than 5 µm. The posterior end was thick and occasionally had ridges (e.g. Figs. 1b–d, 2g), which were rounded and smooth and occasionally had one or more pointed adhesive uroids (Fig. 1f) or a larger knobby uroid. The granuloplasm represented most of the area of the amoebae cells, and contained large amounts of refractive granules, vacuoles, the cell nucleus and normally a single parasome (from one to three were seen). The parasomes varied in size (5–90 µm^2^) within, and among, isolates (Table 2). In the majority of amoebae, regardless of isolate, the parasomes were oval and similar in size (mean 3.2–5.5 µm in diameter), but in some individual amoebae (Fig. 1a, b, d) they were rounded and much larger (7.9–10.9 µm in diameter). The occasional occurrence of such large parasomes did not appear to be strain related. The parasome was always in close contact with the amoeba nucleus, and this pair was normally located in the anterior half of the granuloplasm. Nuclei were vesicular, with a rounded and more distinct karyosome, averaging 2.7 µm in diameter.

**Table 2:** *Paramoeba perurans* trophozoite length, width and size (*Cell area*) and diameter of the nuclei and parasomes (*Perkinsela amoeba*-like organism; *PLO*) for the 10 isolates included in this study

Isolate	*n*	Cell length (µm)		Cell width (µm)			Cell area (µm^2^)			Nucleus diameter (µm)			PLO diameter (µm)		
		Mean (SD)	Range	Mean (SD)	Range		Mean (SD)	Range		*n*	Mean (SD)	Range	*n*	Mean (SD)	Range
1	31	23 (4)	15–34	34 (6)	23–47		537 (121)	354–790		9	6.3 (0.8)	5.1–7.6	15	5.0 (1.4)	3.6–8.3
2	30	32 (11)	20–56	36 (8)	19–58		706 (144)	439–999		11	7.5 (0.5)	6.9–8.8	16	3.9 (0.9)	3.0–5.6
3	32	29 (7)	17–47	39 (9)	24–64		802 (232)	432–1481		27	6.1 (0.3)	5.3–7.3	19	4.4 (1.3)	2.6–7.9
4	31	24 (6)	17–41	30 (6)	19–41		493 (134)	277–864		17	5.1 (0.8)	4.6–5.8	15	4.6 (1.0)	3.2–6.4
5	30	25 (6)	18–44	30 (6)	21–42		552 (133)	341–961		22	5.8 (0.6)	4.5–7.3	14	4.2 (0.8)	3.1–6.1
6	32	25 (4)	17–36	33 (6)	19–47		583 (151)	388–1291		15	5.3 (0.4)	4.0–6.3	10	3.2 (0.4)	2.5–3.9
7	30	29 (7)	16–50	36 (8)	24–54		764 (190)	443–1182		23	5.7 (0.4)	5.2–6.9	12	5.5 (1.9)	4.0–10.9
8	30	24 (4)	16–35	26 (6)	19–46		453 (113)	307–788		29	5.1 (0.7)	4.5–6.2	16	3.9 (0.4)	3.0–4.6
9	30	30 (10)	15–55	43 (9)	24–57		787 (133)	416–1076		11	6.5 (0.4)	5.4–7.8	10	4.4 (0.7)	3.6–5.8
10	31	27 (4)	19–36	28 (5)	19–37		560 (112)	351–826		18	5.5 (0.8)	4.9–6.4	15	4.1 (0.4)	3.4–4.9
All	307	27		34			624			182	5.7		142	4.3	

There were highly significant differences in amoeba size and shape, and parasome size (in all cases *P* < 0.001) (Table 2). Notably larger amoebae (in area) were seen for isolates 3 and 9, while they were smaller for isolates 4 and 8. The shape of the locomotive form, as represented by their length/width ratio in hanging drop preparations, also varied, as this was high (rounded amoebae) in isolates 8 and 10, and particularly low (wide amoebae) in isolates 1 and 9. The size of measurable nuclei varied significantly, as these were larger in isolates 2 and 9 and particularly small in isolates 5 and 6. Parasome size varied among strains, and isolate 7 had particularly large parasomes (post hoc multiple comparison, *P* < 0.001), while the parasomes in isolate 6 were smaller than those in the other isolates (post hoc multiple comparison, *P* < 0.001). The sizes of the amoebae, parasomes and nuclei did not correlate with culture ‘age’ (*r*_*s*_ < 0.30, *P* > 0.40) for any of the strains, irrespective of how long they had been kept in culture (range 4–59 months).

### Morphology of floating forms

**Fig. 3 a–d:**
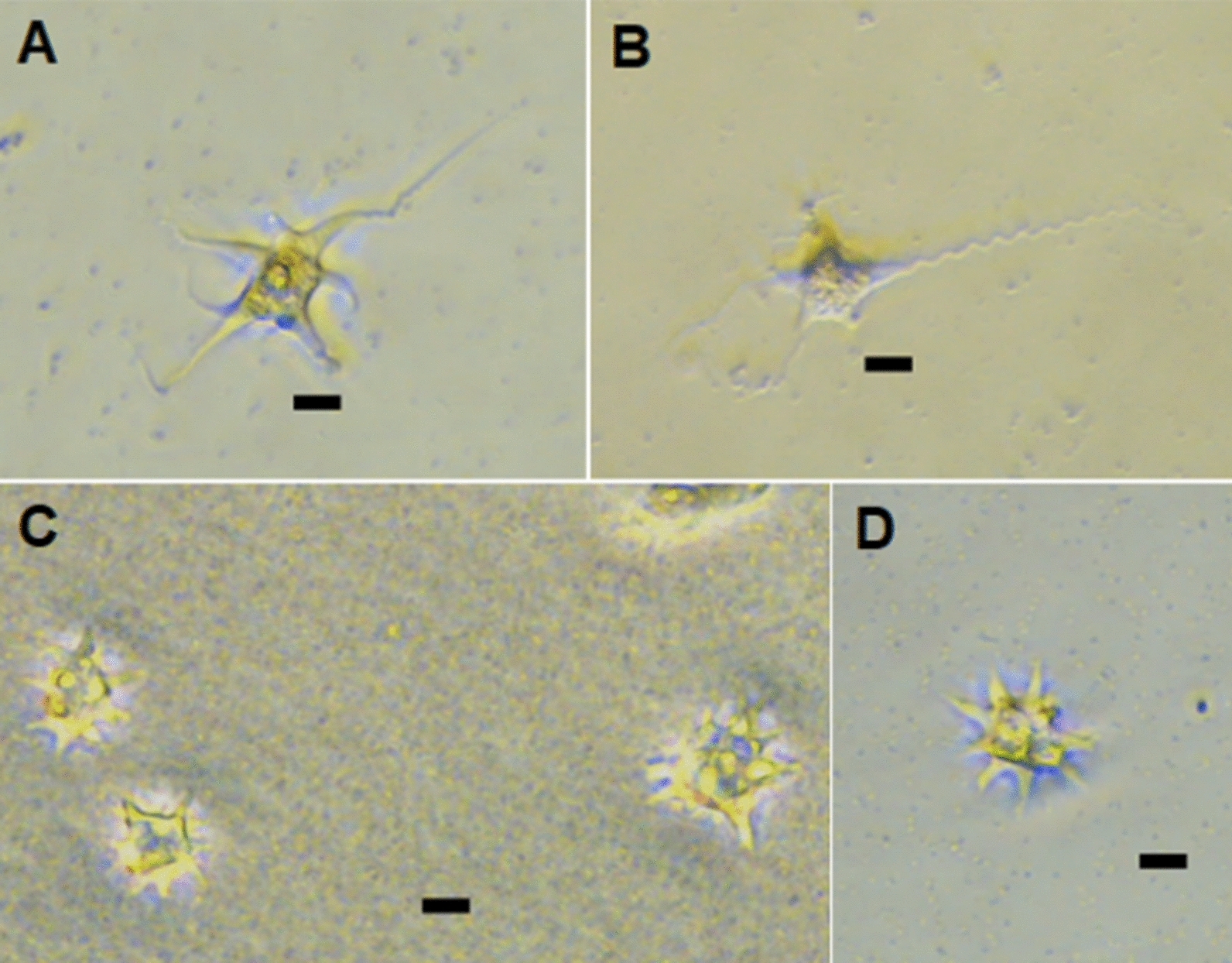
Examples of the morphology of floating *Paramoeba perurans* examined in this study. **a**, **b** Isolate 2; **c**, **d** isolate 1. Scale bars represent 20 µm

Floating forms of the amoebae were observed during the determination of growth curves for all isolates at 15 °C. The floating forms had different morphologies, examples of which can be seen in Fig. 3. The cell body was spherical or subspherical with thin, long (generally 25–30 µm, but reaching 57 µm), sometimes corkscrew-shaped pseudopodia (e.g. isolate 2; Fig. 3a, b) or with shorter tapering, pointed pseudopodia, which gave the amoebae a star-shaped appearance (e.g. isolate 1; Fig. 3c, d). The number of pseudopodia counted varied from one to 14, but the majority of these amoebae had five to six long pseudopodia (pseudopodia length:cell body diameter, range from 2.5:1 to 3:1).

### *Paramoeba perurans* population growth patterns at 15 °C

#### Amoebae attached to the cell plate bottom

**Fig. 4:**
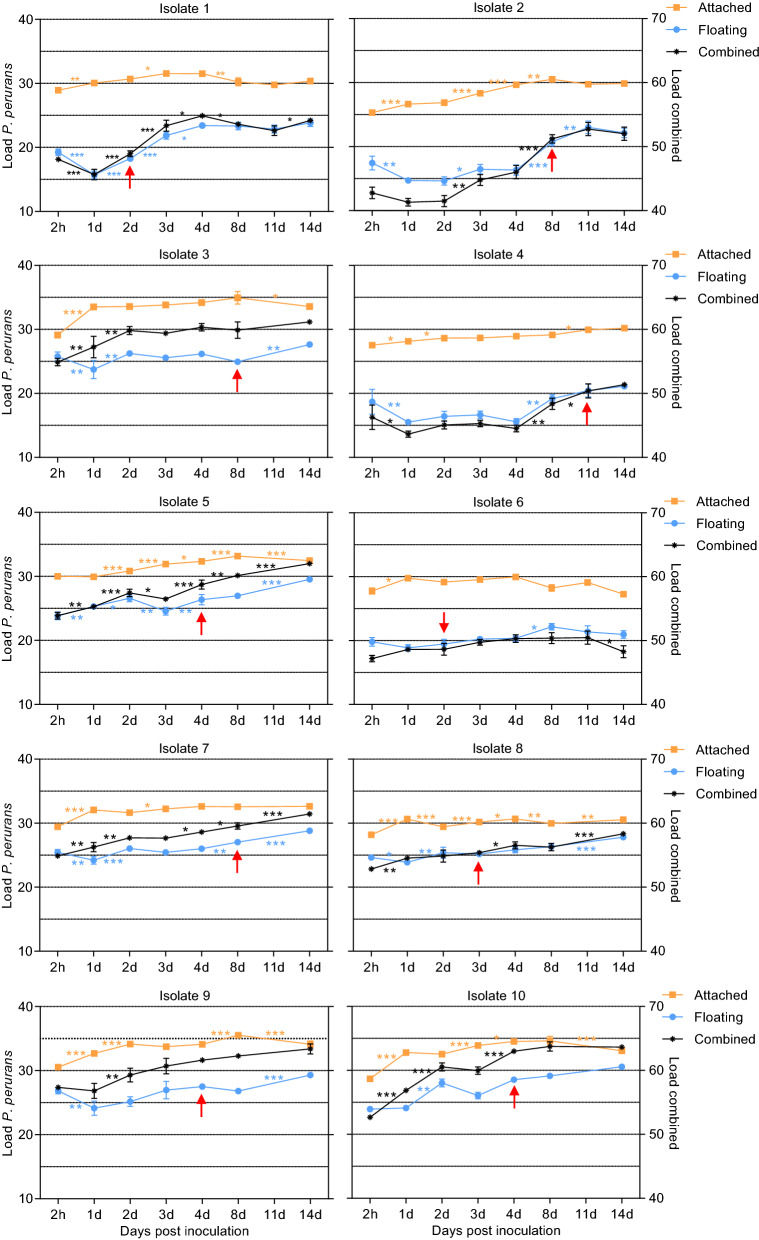
Growth of *Paramoeba perurans* at 15 °C over 14 days. Left-hand *y*-axis denotes *P. perurans* load scales for attached and floating amoebae, right-hand *y*-axis denotes *P. perurans* load scales for floating and attached amoebae combined. Red arrows indicate the first observation of floating amoebae in the preparation. Values are presented as amoeba load (45–Ct value). Error bars indicate SD;* d* day. * *P* < 0.05, ** *P* < 0.01, *** *P* < 0.001

At 15 °C, all isolates except isolate 6 had an increase in overall amoebae population levels from day 1 to day 14. The observed maximum load was not related to the initial amount of amoebae seeded to the wells. All of the isolates, except isolate 5, showed a marked increase in the amounts of attached amoebae from 2 h to 1 dpi (Fig. 4). Some isolates (isolates 1, 3, 5 and 10) showed a gradual increase in the amount of attached amoebae from day 1 until a plateau was reached (3-8 days), followed by a decrease in attached amoebae (4-14 days). Isolate 7 showed a gradual increase to day 3 and thereafter no change, isolate 4 a gradual increase throughout, and isolate 6 a decrease from 1 to 14 dpi. Isolate 8 had an irregular growth pattern with peaks at 1, 4 and 14 dpi. Most isolates (isolates 2, 3, 5, 9 and 10) had reached a plateau or maximum density by day 8 after inoculation. Three isolates (isolates 1, 6 and 7) reached a plateau earlier, i.e. after 1–3 days.

#### Amoebae in suspension (floating amoebae)

An initial decrease in the amount of free-floating amoebae was seen in most isolates from 2 h to 1 day (isolates 1–4, 7–9). In isolate 5 there was an increase, while no significant change was detected in isolates 6 and 10. The overall pattern was an increase from day 1 to 8. In several isolates a marked further increase occurred from day 8 to 14 (isolates 2, 3, 5, 7, 8, 9), often coinciding with a decrease in attached amoebae (Fig. 4). The appearance of the floating morph occurred at different time points for the various isolates (Fig. 4), and there were large differences in the numbers produced. Based on microscopic observations and real-time RT-PCR data, it seemed that the floating morphs appeared at around the same time that the plateau phase of the attached amoebae was reached, which varied from 2 to 8 days. For most isolates (1, 5, 6, 8–10), floating morphs were detected early in the experiment (2–4 dpi), but for isolates 2, 3 and 7, these were not seen until 8 dpi. For isolate 4, some floating amoebae were seen at 1 dpi, but were not observed at 2–8 dpi, while new observations of floating forms were recorded from 11 dpi and onwards.

#### Locomotive amoebae in water surface microlayers

**Fig. 5:**
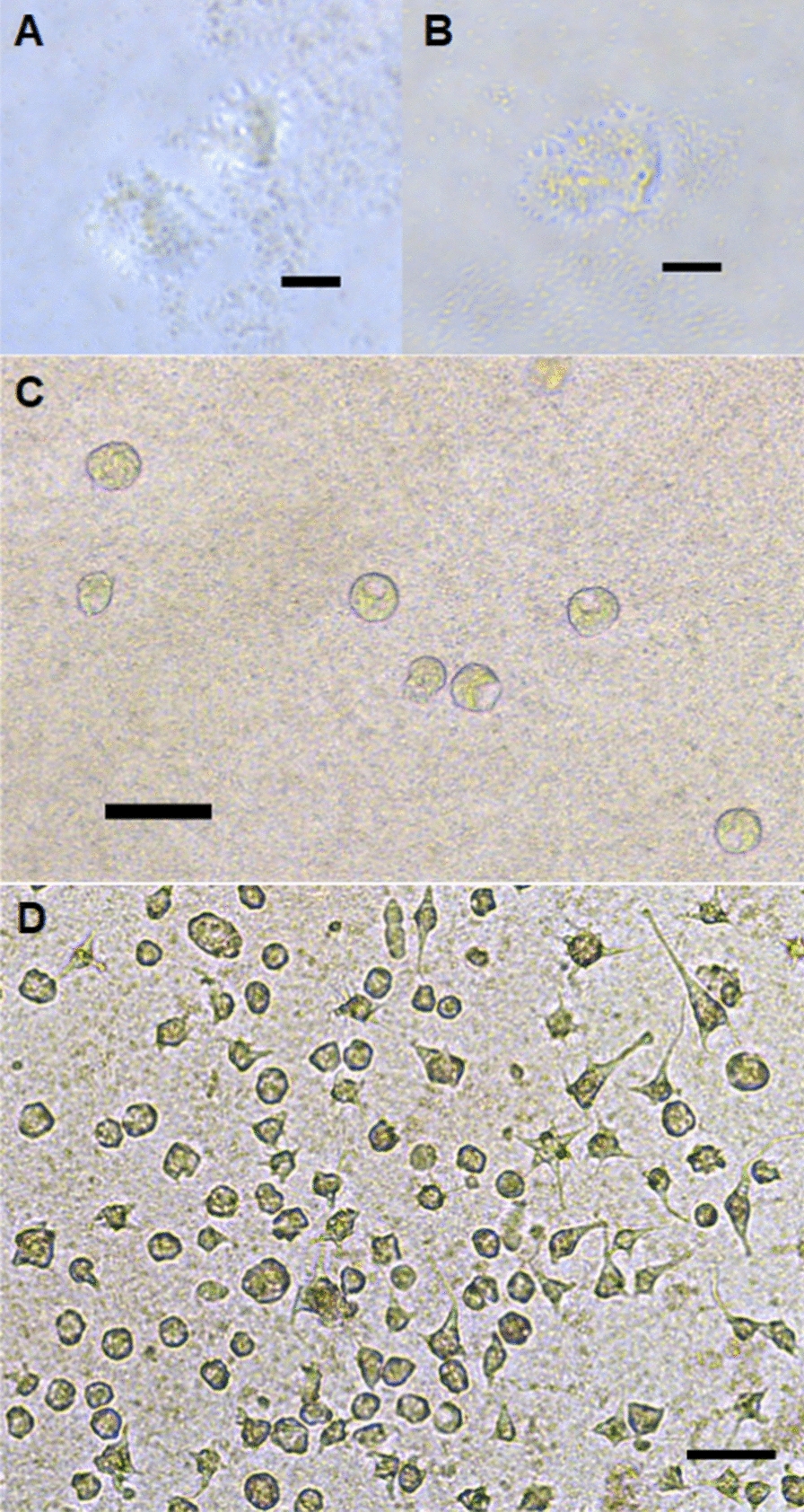
Morphology of attached *Paramoeba perurans* of isolate 4 in the surface microlayer (**a**, **b**), floating amoebae grown at 4 °C (**c**) and isolate 5 grown at 21 °C, at 8 days post-inoculation (**d**). Scale bars represent 20 µm (**a**, **b**) and 50 µm (**c**, **d**)

Interestingly, for some isolates, especially isolate 4, large amounts of the locomotive morph could be observed in the surface microlayer of the wells (Fig. 5a, b), especially from day 8 onwards. Locomotive amoebae were also detected in the surface microlayers for isolates 1, 2 and 6, but in lower numbers (not quantified).

### *Paramoeba perurans* population growth at different temperatures

#### Population growth at 4 °C

**Fig. 6:**
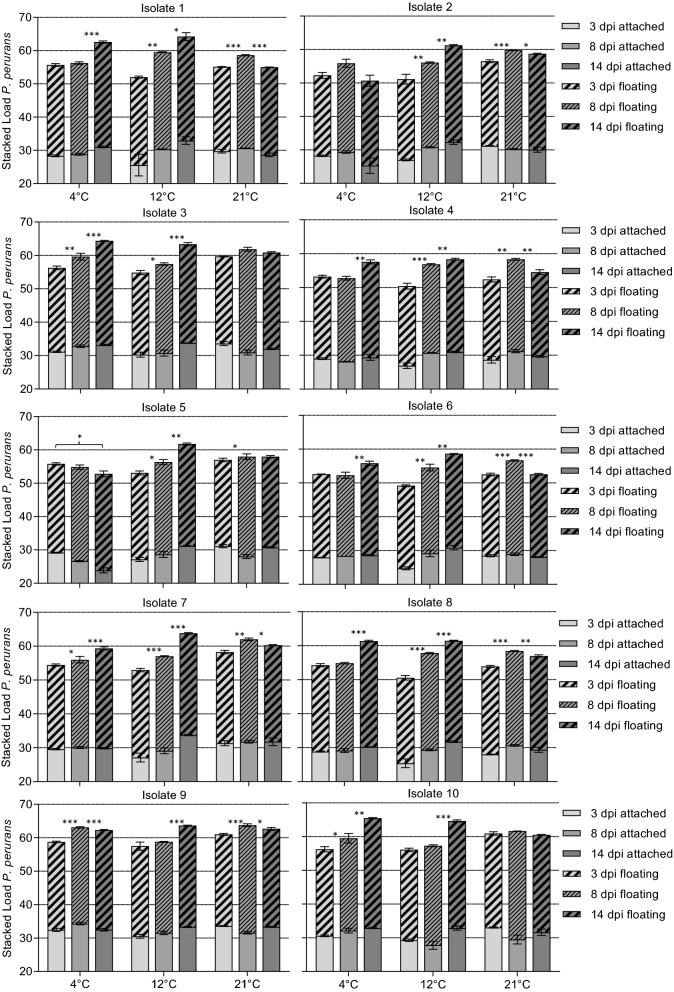
Growth of the different *Paramoeba perurans* isolates cultured at 4, 12 and 21 °C. Values are presented as amoeba load (45 – Ct value).* dpi* Days post-inoculation. Error bars indicate SD. * *P* < 0.05, ** *P* < 0.01, *** *P* < 0.001

Isolate 5 was the only isolate that showed a decline in amoebae levels at each sampling at 4 °C, although isolate 2 also showed an overall reduction in amoebae levels throughout the experiment (Fig. 6). An increase in *P. perurans* levels was seen for all other isolates, with the highest load either at 8 dpi (isolates 2 and 9) or 14 dpi (isolates 1, 3, 4, 6–8 and 10). The morphology of floating amoebae was usually different at 4 °C from that observed at higher temperatures (free floating morphs) in that their pseudopodia were very short or absent (Fig. 5c). At 14 dpi there were large differences in the numbers of amoebae between the clonal isolates.

For the long-term survival study of amoebae in cell culture flasks (< 8 weeks at 4 °C), attached forms were seen in all triplicate flasks after 1 week of subculturing after being kept for 5 weeks (37 days) at 4 °C. The amoebae numbers in the cell culture flasks were low at this time (8 days post-transfer from 4 °C to 15 °C), with ~ 15 attached amoebae per flask when counted under a microscope, indicating that the amoebae were in a restitutive phase, as higher numbers would be expected under standard growth conditions. Three weeks after the subculturing (20 days post-transfer from 4 °C to 15 °C), the growth in the cell culture flasks had been restored to normal amoebae levels (compared to maintenance cultures). When amoebae were collected from the cell culture flasks held at 4 °C at 8 weeks (57 dpi), no viable amoebae were observed when examined at 8 or 20 days after subculturing (post-transfer from 4 °C to 15 °C).

#### Population growth at 12 °C

All isolates showed an increase in overall amoebae levels at each sampling when grown at 12 °C. For isolates 1–3 and 5–9 the attached amoebae population levels increased at each sampling. For isolate 4 the attached amoebae levels were similar at 8 and 14 dpi, while isolate 10 showed the first significant (Newman-Keuls, *P* < 0.001) increase 14 dpi. For all isolates, the floating amoebae levels increased.

At 12 °C, growth (load) did not correlate with age (*r*_*s*_ < 0.31, *P* > 0.38) for the different strains, which had been kept in culture for 1–25 months (age).

#### Population growth at 21 °C

When the amoebae were grown at 21 °C the highest densities were seen at 8 dpi for all isolates (attached and floating amoebae combined). Both attached and floating forms of amoebae could be observed at all sampling points for all isolates, but at the samplings performed at 8 and 14 dpi, the cultures appeared to be in poor condition, as a large proportion of the observed amoebae were round in shape and did not have pseudopodia (Fig. 5d).

### Growth and survival at different levels of salinity

**Fig. 7:**
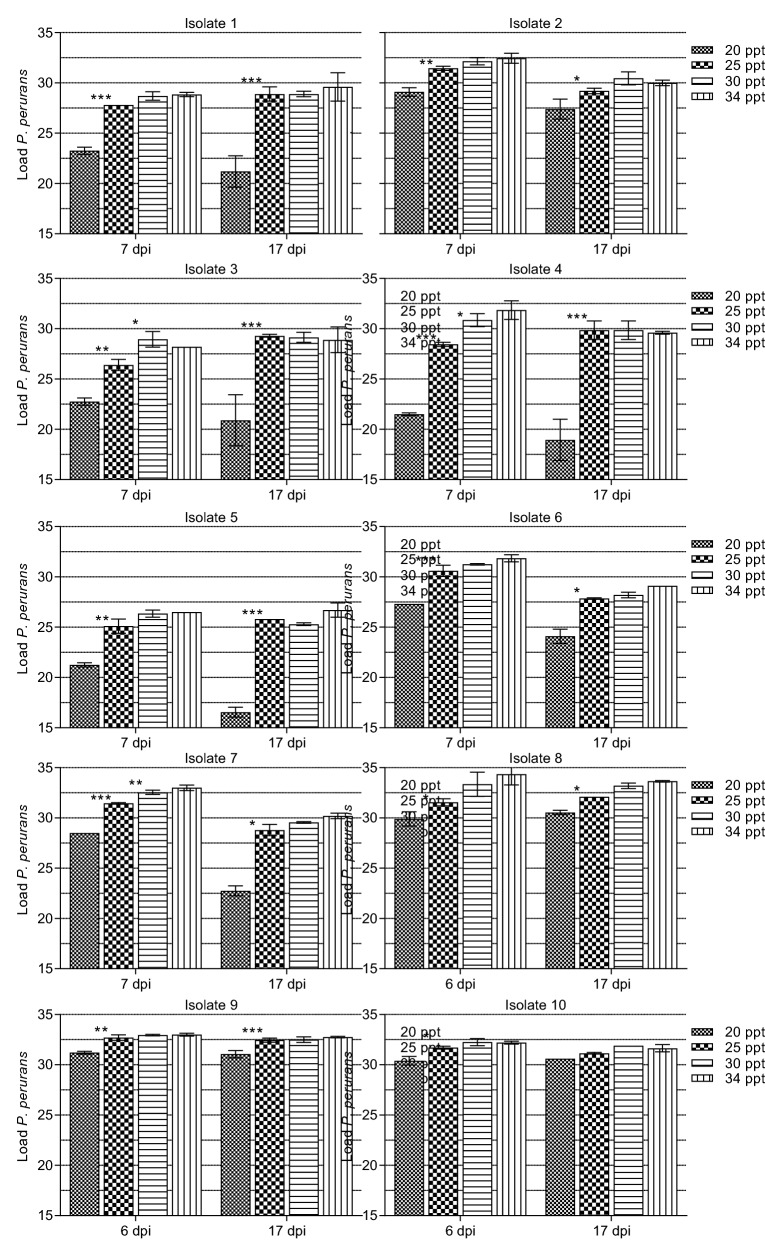
Growth of 10 different isolates of *Paramoeba perurans* at four different salinities [20, 25, 30 and 34 ‰ (parts per thousand;* ppt*)]. Samples were collected after 6 (isolates 1–7) or 7 days (isolates 8–10), and after 17 days. Values are presented as amoeba load (45 – Ct value). Error bars indicate SD. * *P* < 0.05, ** *P* < 0.01, *** *P* < 0.001

All amoeba isolates were exposed to four different salinities (20, 25, 30 and 34 ‰) for 20 days to assess whether there were any variations in tolerance or preference for seawater at reduced salinity (Fig. 7).

At the first sampling point, 6 dpi (experiment 2, isolates 8–10) or 7 dpi (experiment 1, isolates 1–7), the best growth (highest load) was seen at the highest salinities (30–34 ‰) for all of the isolates. Amoebae densities at these highest salinities did not differ significantly. All of the isolates had reduced growth at 20 ‰ compared to the other salinities, and 20‰ was the only salinity that had an effect on growth across isolates (ANOVA, *F*_*3,67*_ = 100.4, *P* < 0.001; Fig. 7). Only isolates 3, 4 and 7 had significantly reduced amoebae densities at 25‰.

At the second sampling, 17 dpi, the only salinity affecting growth across isolates was 20‰ (ANOVA, *F*_3,67_ = 71.5, *P* < 0.001). An overall slight yet significant reduction of load was seen at 6–7 dpi to 17 dpi (ANOVA *F*_1,145_ = 19.0, *P* < 0.001; Fig. 7). The largest decrease at 20‰ was seen for isolates 4 and 5 at 17 days (− 10.9 and − 9.3 Ct values, respectively), while in isolates 9 and 10, the effect of this salinity was marginal (− 1.4 and − 0.5 Ct values, respectively). Across salinities, load varied highly significantly with isolate (ANOVA, *F*_3,67_ = 68.8 and 39.5, at 6–7 and 17 dpi, respectively; both *P* < 0.001). Isolate 5 showed particularly poor growth (as load), while isolate 8 showed the best growth.

Even though we observed very few attached amoebae in the 20‰ wells by microscopy after 20 days, all isolates except isolate 5 survived exposure to these unfavourable conditions, as growth recommenced when the amoebae were transferred back to full-strength seawater (34‰). The varying periods of time between isolation of the amoebae isolates and the experiment were not seen to have any impact on the measured growth under reduced salinity (20‰; *r*_*s*_ < 0.36, *P* > 0.31).

### AGD-challenge study

In Atlantic salmon challenged with *P. perurans* (isolate 2) at 34‰ for 1 h and transferred to 2 ‰ or 34‰, no infection was observed in fish grown on at 25‰ for up to 61 days. Real-time RT-PCR analyses for *Paramoeba* sp. from gill samples taken at 15, 27 and 61 dpc were negative, and no amoebae were re-isolated on agar. In the 34 ‰ group, however, two out of five fish were positive at day 15 (Ct values of 32.6–32.8, mean gill score of 1), and five out of five at 27 dpc (Ct values of 23.4–27.1, mean gill score of 3.2). Fish mortality was observed in the 34‰ tank at 42 (one fish), 45 (one fish) and 57 dpc (two fish), while the challenge in the tank at 25‰ was terminated at 61 dpc after additional sampling of fish from this tank. No fish mortality was observed in the 25‰ tank. The fish that died at 42–57 dpc in the 34‰ tank had gill scores between 4 and 5 and were all *Paramoeba* sp. positive, with Ct values in the range of 18.1–24.8.

## Discussion

Differences existed between the clonal isolates in their morphology, growth curves and temperature and salinity preferences. Some of the isolates (isolate 2–5 and 8) described in this study have been used in challenge studies [5, 13, 18, 42], with varying results regarding disease development. More specifically, isolates 2, 4, 5 and 8 were shown to be highly virulent for salmon, inducing severe AGD, whereas isolate 3 appeared to be less virulent. Differences in virulence between clonal isolates, both in vitro and in vivo, have been reported, but the mechanisms underlying these variations have not been documented. Moreover, as pointed out by Collins et al. [17], such differences in virulence, even if stable at the genomic level, may not be stable at the gene expression level. Bridle et al. [16] reported that a clonal culture that was shown to be virulent after 70 days of culture lost virulence after 3 years in culture (> 200 passages). At the time of this study, the virulent isolate 2 (H02/13Pp) had been virulent for 9 years (personal observation), since its initial isolation in 2013. Variation in the period of time between isolation and the experiments was not found to have any impact on the measured isolate characteristics. Virulence may relate to the production of extracellular products or factors that influence growth rate [14], amoebae numbers, or resistance to elimination mechanisms exhibited by the host [17]. Extracellular products may degrade tissues directly or destroy protective barriers [43]. Research on *P. perurans* is complicated by the present inability to grow the amoeba in axenic cultures. It is currently not known how virulence in cultured amoebae may vary over time. 

Research has broadened in recent years to include examination of the role of bacteria in xenic amoeba cultures and how they may influence *P. perurans*’ virulence [14, 35]. The role of bacteria may be symbiotic, such that they produce compounds that facilitate *P. perurans*’s growth, or they may be a food source for the amoeba. Various marine bacteria have been reported to live in close association with *P. perurans*, with both* Vibrio* spp. [35] and ‘*Candidatus* Syngnamydia salmonis’ [21] having been detected intracellularly. The former may be prey, while the latter appears to be a symbiont characteristically present in most isolates. Strikingly, nine out of the 10 isolates that we examined in this study harboured ‘*Candidatus* Syngnamydia salmonis’ [21]. To date, we have only identified two clonal *P. perurans* isolates without the ‘*Candidatus* Syngnamydia salmonis’ symbiont: isolate 2 (H02/13Pp C2), and an isolate from Scotland (SCO01/18 F3). Since isolate 2 lacks ‘*Candidatus* Syngnamydia salmonis’ but is highly virulent (personal observation), it seems unlikely that the latter has a major impact on its virulence. 

The ultrastructural features of *Paramoeba* spp. under in vitro conditions have been described [33, 44, 45]. In 2007 it was shown that *P. perurans* was the aetiological agent of AGD [3], and protocols for its long-term in vitro culturing were published in 2012, facilitating further research [2]. Since then, several studies of *Paramoeba* spp. describing cell morphology of the amoebae in culture have been conducted using light microscopy together with transmission and scanning electron microscopy [1, 13, 21, 23, 28, 46, 47]. Only a few of these studies reported that the experiments were conducted with clonal amoeba cultures [13, 21, 23, 46], which are necessary to obtain species strain- or genotype-specific information. The descriptions from morphological examination of the 10 clonal *P. perurans* isolates in this study agree with previous descriptions of *Paramoeba* spp. with respect to size and morphological features of both the attached and floating forms. In the first published description of *P. perurans*, Young et al. [3] noted that its size was 41–56 µm in adherent form, the nucleus was 3.3–6.0 µm and the parasomes 5.3–8.0 µm. Similar sizes have also been reported for *P. perurans* more recently, i.e. 15–40 µm [14] and 10–20 µm [35]. Karlsbakk et al. [6] reported that free amoebae from infected gills measured from 17.3 to 32.5 µm in wet mount preparations, and 22.4–28.5 µm in culture. The attached forms can exceed 50 µm in size [48]. The greatest sizes of attached amoebae of the 10 different clones measured in the present study were between 26 and 43 µm (range 19–64 µm). The karyosomes measured up to 2.7 µm in diameter. All of the isolates examined had one or more (up to three) parasomes with a mean diameter of 3.2–5.5 µm, which were always located close to the nucleus; the size and shape of most of the parasomes were in agreement with previous descriptions. In a few amoebae, however, the parasome was larger (7.9–10.9 µm), perhaps indicating pre-division forms. The clonal isolates (attached amoeba) varied in size, and the mean size (area) ranged from 453 to 802 µm^2^. Dykova et al. [44] noted intra-strain size differences within 15 clonal strains of *P. pemaquidensis* and three clonal strains of *P. branchiphila*.

Although genetically indistinguishable (with respect to partial 18S rDNA sequences), the clonal isolates of *P. perurans* in our study had different phenotypes in both the attached forms (with respect to size) and the floating forms. The appearance of the floating forms in the present study corresponded to previous descriptions of *Paramoeba* [23, 44, 46], but also here differences between strains could be observed, as the shape, numbers, and lengths of pseudopodia together with the shape and size of the cell bodies varied. For instance, clonal isolates 1 and 4 had small lobose pseudopodia, whereas isolate 2 had long, extended, sometimes corkscrew-shaped pseudopodia. The presence of a pointed adhesive uroid in some amoebae was noted for three isolates. Such structures have not previously been noted in symbiotic *Paramoeba* spp. of fish, but are known to occur in other species of this genus, such as *Paramoeba atlantica* and *Paramoeba longipodia* [28, 49].

For clonal isolate 4, large amounts of locomotive forms of the amoeba were also seen in the surface microlayer of the wells. Since observing this in the in vitro study presented here, we have also seen locomotive amoebae in these layers in several *P. perurans* cultures. Hence this phenomenon has now been observed in a range of isolates, and it appears to be a general trait that is not related to strain. Amoebae move and feed using their pseudopods, and it has been suggested that feeding (population growth) in *Paramoeba* may only occur in the attached or locomotive form [33, 48, 50, 51], while in the floating form the long, thin pseudopods (filopodia) contribute to buoyancy and likely also to attachment to new substrates (e.g. hosts). In *Acanthamoeba* spp*.*, attached forms of amoebae in the surface microlayers may allow them to graze and phagocytose bacteria while being transported passively over enormous distances; the surface microlayers are known to be a micro-niche that is especially rich in nutrients and microorganisms [52]. To the best of our knowledge, this is the first report to show that *P. perurans* may form grazing trophozoites in the surface microlayer of water, and it consequently seems possible that *P. perurans* can survive and spread long distances in this way.

Collins et al. [12] observed optimal growth of non-clonal *P. perurans* populations held in vitro at 15 °C and a salinity of 35‰, with amoebae numbers doubling every 14 h. Their findings agree well with those of our experiments in which a culturing regime was used that has been sustained since October 2013 at our facility, where clonal cultures of *P. perurans* have been kept in culture for > 9 years at 15 °C and split every 14th day (personal observations). Collins et al. [12] found that *P. perurans* were able to grow at 20–50 ‰ at 15 °C, and at 25–50 ‰ at temperatures of 8, 11 and 18 °C. The lower temperature and salinity limits for *P. perurans* population growth have been reported to be between 4 and 8 °C and between 20 and 25‰ [12]. Although no growth was detected at 4 °C in that study, amoeba populations were shown to remain stable over the experimental period of 15 days at 2–4 °C [12]. In our study, an increase in *P. perurans* levels from day 3 to day 14 was detected for eight out of the 10 isolates when held at 4 °C. *P. perurans* (isolate 2) was also shown to be viable after being kept in cell culture flasks for 5 weeks at 4 °C. It is interesting to note that, in the temperature study at 4 °C, the pseudopods of the floating amoeba were very short or retracted; this probably led to a reduction in surface area as a response to environmental stress, in agreement with Lima et al. [19]. At temperatures and salinities below growth permissive levels, the number of attached amoebae relative to amoebae in suspension was lower [12]. The same was true here for cultures held at 21 °C. In agreement with Collins et al. [12], this may have been related to environmental stress in the enclosed in vitro set-up (e.g. a build-up of toxic metabolites or the depletion of prey organisms).

Growth of the various isolates in our study was examined at 4, 12, 15 and 21 °C. At 15 °C, the attached forms of various isolates were observed to reach a plateau phase at different time points after inoculation into wells. The plateau phase ([12]) was reached approximately at the same time point that the floating forms could be identified in the various cultures, as previously reported by Collins et al. [12]. Floating amoebae were observed between 3 and 14 (mean 5.4) dpi for the various isolates. After the plateau phase, a reduction in the attached amoeba levels could be seen for most isolates, and at the same time an increase in the floating amoebae levels. The amoebae population levels detected for the various cultures also varied between clonal isolates. The highest levels of amoebae were seen for attached amoebae from isolate 9 at 8 dpi, as amoebae proliferate until floating forms start to appear due to increased density in culture. For floating amoebae, the highest levels were seen at 14 dpi for isolate 10. The time points where floating forms were seen were not related to variation in the starting numbers of amoebae (higher in isolates 1, 2, 4 and 6). Also, the doubling time for *P. perurans* held at 15 °C and 35‰ has been reported to be only 14 h [12], so the variation in starting numbers of amoebae was thought to have had only a minor influence. Indeed, the highest densities of amoebae at 15 °C were seen for the other isolates with lower starting amounts (e.g. 3, 5, 7, 9, 10). At the highest temperature, 21 °C, amoebae had a similar appearance to amoebae grown under standard growth conditions (15–16 °C), but there were more small, rounded amoeba (‘pseudocysts’), which indicated that the culture conditions were suboptimal for the amoebae. High amounts of small, rounded amoeba are often seen in dense cultures or when subculturing has been infrequent [23]. In the present study, cultures with amoeba incubated at 21 °C seemed to stagnate sooner, as the plateau phase was reached at an earlier time point (between 3 and 8 days), with a reduction in attached amoeba numbers seen for most isolates at 8 dpi.

Contractile vacuoles are involved in maintaining osmotic equilibrium in the amoeba under periods of reduced salinity [19]. When the *P. perurans* isolates were exposed to a range of salinities (20–34‰) in our study, there was a reduction in amoeba growth at 20‰, and a general preference for higher salinities was seen. When the amoebae were transferred back to full-strength seawater after 20 days, population growth resumed. These results agree well with those from the in vivo challenge study of Atlantic salmon presented here, where fish were challenged with isolate 2 at 34‰ for 1 h and then split into two groups and exposed to either 25 or 34‰. *P. perurans* infections were not established in the 25‰ tank. Clonal *P. perurans* isolate 2 could not establish an infection in salmon transferred to a lower salinity (25‰) immediately after the challenge, even when held at full salinity during the 1-h challenge. In contrast, in the tank at 34 ‰ salinity, most of the fish were infected with *P. perurans* (real-time RT-PCR positive), with some fish attaining a gill score of 4–5. In contrast, clonal isolate 3 was incidentally isolated from a non-AGD-challenge experiment with fish held at 25‰ [38], and the amoeba strain was shown to proliferate at this salinity—although at low levels and with low prevalence. A possible explanation for this is that some amoebae in cases of late stage AGD are better ‘protected’ than amoebae during the initial phase of infection (due to excess mucus and persistent cavitation) [12]. In our in vitro salinity study, isolate 3 was one of three isolates that also showed reduced growth at 25‰ at 6–7 dpi. 

AGD outbreaks may vary. The disease mostly develops to a treatable level, although it may progress to cause mortality of the most severely affected individuals, or it may clear over time with a change in salinity or season. Salmon are usually treated when gill scores reach ≥ 2 in 30% of the examined population [53], although there has been a shift to more proactive treatments at lower mean gill score. When the mean gill score of fish reaches 3, there are few gill surfaces left without mucus patches [54]. When culturing *P. perurans*, we observed phenotypical differences such as size differences and the appearance of floating forms of the amoeba. At present, treatment decisions do not take into consideration the type of isolate that has been detected, as no phenotypic traits have been linked to virulence, and the virulence mechanisms are not understood. The composition of the bacterial communities associated with the clonal isolates, and how this might change with exposure to various salinities and temperatures, is clearly of great interest but was not examined in the present study. If a link is found between specific isolates and virulence, identifying which isolate has infected a fish population may help in deciding whether to treat the fish or not.

## Conclusions

We have described the light microscopic characteristics of 10 clonal isolates of *P. perurans*, and investigated their growth characteristics under different salinities and temperatures. Variation in morphology and size between isolates agreed with observations from previous studies. The observed differences between some of the isolates with respect to their temperature and salinity preferences may be of importance for mitigation strategies. The relationship between bacteria and the proliferation and morphology of *P. perurans* and the development and virulence of AGD should be investigated further.

## Supplementary Information

**Additional file 1**: Alignment A1. Alignment of the partial 18S rRNA sequence (736 base pairs) from the 10 clonal cultures of *Paramoeba perurans* against EF216904 (from Young et al. [3]).

## Data Availability

The datasets used and/or analysed during the current study are available from the corresponding author on reasonable request. Voucher series (5 per isolate) from all 10 amoebae isolates in 96% ethanol are stored at the University of Bergen, Norway. These are available from the authors (LA, EK) on request.

## References

[1] Feehan CJ, Johnson-Mackinnon J, Scheibling RE, Lauzon-Guay J-S, Simpson AG (2013). Validating the identity of *Paramoeba invadens*, the causative agent of recurrent mass mortality of sea urchins in Nova Scotia, Canada. Dis Aquat Organ.

[2] Crosbie PB, Bridle AR, Cadoret K, Nowak BF (2012). In vitro cultured *Neoparamoeba perurans* causes amoebic gill disease in Atlantic salmon and fulfils Koch’s postulates. Int J Parasitol.

[3] Young N, Crosbie P, Adams M, Nowak B, Morrison R (2007). *Neoparamoeba perurans* n. sp., an agent of amoebic gill disease of Atlantic salmon (*Salmo salar*). Int J Parasitol..

[4] Crosbie P, Ogawa K, Nakano D, Nowak B (2010). Amoebic gill disease in hatchery-reared ayu, *Plecoglossus altivelis* (Temminck & Schlegel), in Japan is caused by *Neoparamoeba perurans*. J Fish Dis.

[5] Haugland GT, Olsen A-B, Rønneseth A, Andersen L (2017). Lumpfish (*Cyclopterus lumpus* L.) develop amoebic gill disease (AGD) after experimental challenge with *Paramoeba perurans* and can transfer amoebae to Atlantic salmon (*Salmo salar* L.). Aquaculture..

[6] Karlsbakk E, Olsen AB, Einen A-CB, Mo TA, Fiksdal IU, Aase H (2013). Amoebic gill disease due to *Paramoeba perurans* in ballan wrasse (*Labrus bergylta*). Aquaculture.

[7] Young ND, Dyková I, Snekvik K, Nowak BF, Morrison RN (2008). *Neoparamoeba perurans* is a cosmopolitan aetiological agent of amoebic gill disease. Dis Aquat Organ.

[8] Oldham T, Rodger H, Nowak BF (2016). Incidence and distribution of amoebic gill disease (AGD)—an epidemiological review. Aquaculture.

[9] Mitchell S, Rodger H (2011). A review of infectious gill disease in marine salmonid fish. J Fish Dis.

[10] Nowak B, Valdenegro-Vega V, Crosbie P, Bridle A (2014). Immunity to amoeba. Dev Comp Immunol.

[11] Munday B, Foster C, Roubal F, Lester R (1990). Paramoebic gill infection and associated pathology of Atlantic salmon, *Salmo salar*, and rainbow trout, *Salmo gairdneri* Tasmania. Pathol Marine Sci.

[12] Collins C, Hall M, Fordyce MJ, White P (2019). Survival and growth in vitro of *Paramoeba perurans* populations cultured under different salinities and temperatures. Protist.

[13] Nylund A, Røed M, Blindheim S, Trösse C, Andersen L (2021). Experimental challenge of Atlantic salmon *Salmo salar* using clones of *Paramoeba perurans*, P. *pemaquidensis* and *Tetramitus* sp.. Dis Aquat Organ..

[14] Benedicenti O, Secombes C, Collins C (2019). Effects of temperature on *Paramoeba perurans* growth in culture and the associated microbial community. Parasitology.

[15] Botwright NA, Rusu A, English CJ, Hutt O, Wynne JW (2020). A high throughput viability screening method for the marine ectoparasite *Neoparamoeba perurans*. Protist.

[16] Bridle AR, Davenport DL, Crosbie PB, Polinski M, Nowak BF (2015). *Neoparamoeba perurans* loses virulence during clonal culture. Int J Parasitol.

[17] Collins C, Hall M, Bruno D, Sokolowska J, Duncan L, Yuecel R (2017). Generation of *Paramoeba perurans* clonal cultures using flow cytometry and confirmation of virulence. J Fish Dis.

[18] Dahle OMV, Blindheim SH, Nylund A, Karlsbakk E, Breck O, Glosvik H (2020). Atlantic salmon *Salmo salar* and ballan wrasse *Labrus bergylta* display different susceptibility to clonal strains of *Paramoeba perurans*. Dis Aquat Organ.

[19] Lima P, Taylor R, Cook M (2016). Involvement of contractile vacuoles in the osmoregulation process of the marine parasitic amoeba *Neoparamoeba perurans*. J Fish Dis.

[20] Lima P, Taylor R, Cook M (2017). Pseudocyst formation in the marine parasitic amoeba *Neoparamoeba perurans*: a short-term survival strategy to abrupt salinity variation. J Fish Dis.

[21] Nylund A, Pistone D, Trösse C, Blindheim S, Andersen L, Plarre H (2018). Genotyping of *Candidatus* Syngnamydia salmonis (Chlamydiales; Simkaniaceae) co-cultured in *Paramoeba perurans* (Amoebozoa; Paramoebidae). Arch Microbiol.

[22] Tröße C, Kindt M, Blindheim S, Andersen L, Nylund A (2021). Method for cryopreservation of *Paramoeba perurans*. J Fish Dis.

[23] Wiik-Nielsen J, Mo T, Kolstad H, Mohammad S, Hytterød S, Powell M (2016). Morphological diversity of *Paramoeba perurans* trophozoites and their interaction with Atlantic salmon, *Salmo salar* L., gills. J Fish Dis..

[24] Young ND, Dyková I, Crosbie PB, Wolf M, Morrison RN, Bridle AR (2014). Support for the coevolution of *Neoparamoeba* and their endosymbionts* Perkinsela amoebae*-like organisms. Eur J Protistol.

[25] Page FC. Marine gymnamoebae: Institute of Terrestrial Ecology; 1983.

[26] Smirnov A, Nassonova E, Berney C, Fahrni J, Bolivar I, Pawlowski J (2005). Molecular phylogeny and classification of the lobose amoebae. Protist.

[27] Smirnov A (2008). Amoebas, lobose.Encyclopedia of Microbiology.

[28] Volkova E, Kudryavtsev A (2017). Description of *Neoparamoeba longipodia* n. sp. and a new strain of *Neoparamoeba aestuarina* (Page, 1970) (Amoebozoa, Dactylopodida) from deep-sea habitats. Eur J Protistol..

[29] Volkova E, Mishagin D, Kudryavtsev A (2022). Intraspecific variability of* Neoparamoeba pemaquidensis*. SSRN J.

[30] Dyková I, Fiala I, Pecková H (1980). *Neoparamoeba* spp. and their eukaryotic endosymbionts similar to* Perkinsela amoebae* (Hollande), coevolution demonstrated by SSU rRNA gene phylogenies. Eur J Protistol.

[31] Hollande A. Identification du parasome (Nebenkern) de* Janickina pigmentifera* à un symbionte (*Perkinsiella amoebae* nov gen-nov sp.) apparenté aux flagellés Kinetoplastidiés. 1981.

[32] Dyková I, Fiala I, Lom J, Lukeš J (2003). *Perkinsiella* amoebae-like endosymbionts of *Neoparamoeba* spp., relatives of the kinetoplastid* Ichthyobodo*. Eur J Protistol.

[33] Dyková I, Figueras A, Peric Z (2000). *Neoparamoeba* Page, 1987: light and electron microscopic observations on six strains of different origin. Dis Aquat Organ.

[34] Alexander K, Ekaterina V, Fyodor V (2021). A checklist of Amoebozoa species from marine and brackish-water biotopes with notes on taxonomy, species concept and distribution patterns. Protistology.

[35] MacPhail DPC, Koppenstein R, Maciver SK, Paley R, Longshaw M, Henriquez FL (2021). Vibrio species are predominantly intracellular within cultures of *Neoparamoeba perurans*, causative agent of amoebic gill disease (AGD). Aquaculture.

[36] Horn M, Wagner M, Müller KD, Schmid EN, Fritsche TR, Schleifer KH, Michel R (2000). *Neochlamydia hartmannellae* gen. nov., sp. nov. (Parachlamydiaceae), an endoparasite of the amoeba* Hartmannella vermiformis*. Microbiology.

[37] Steinum T, Kvellestad A, Rønneberg L, Nilsen H, Asheim A, Fjell K (2008). First cases of amoebic gill disease (AGD) in Norwegian seawater farmed Atlantic salmon, *Salmo salar* L., and phylogeny of the causative amoeba using 18S cDNA sequences. J Fish Dis.

[38] Smørås C. Betydning av Paranucleospora theridion og Salmonid Alfavirus (SAV) for utvikling av sykdom hos Atlantisk laks (*Salmo salar* L.)-Dobbelsmitte av laks med *P. theridion* og SAV, og betydning av temperatur for densitet av *P. theridion* i laks: The University of Bergen; 2014.

[39] Gunnarsson G, Karlsbakk E, Blindheim S, Plarre H, Imsland A, Handeland S (2017). Temporal changes in infections with some pathogens associated with gill disease in farmed Atlantic salmon (*Salmo salar* L). Aquaculture.

[40] Nylund S, Steigen A, Karlsbakk E, Plarre H, Andersen L, Karlsen M (2015). Characterization of ‘*Candidatus *Syngnamydia salmonis’(Chlamydiales, Simkaniaceae), a bacterium associated with epitheliocystis in Atlantic salmon (*Salmo salar* L.). Arch Microbiol.

[41] Olsvik PA, Lie KK, Jordal A-EO, Nilsen TO, Hordvik I (2005). Evaluation of potential reference genes in real-time RT-PCR studies of Atlantic salmon. BMC Mol Biol.

[42] Hytterød S, Andersen L, Hansen H, Blindheim S, Poppe T, Kristoffersen A, et al. AGD-behandlingsstrategier-Dose-respons-studier med hydrogenperoksid og ferskvann. Vet Rapp. 2017(10–2017).

[43] Serrano-Luna J, Piña-Vázquez C, Reyes-López M, Ortiz-Estrada G, de la Garza M (2013). Proteases from* Entamoeba* spp. and pathogenic free-living amoebae as virulence factors. J Trop Med.

[44] Dykova I, Nowak BF, Crosbie PB, Fiala I, Peckova H, Adams MB (2005). *Neoparamoeba branchiphila* n. sp., and related species of the genus *Neoparamoeba* Page, 1987: morphological and molecular characterization of selected strains. J Fish Dis.

[45] Kim H-J, Cho J-B, Lee M-K, Huh M-D, Kim K-H (2005). Neoparamoeba sp. infection on gills of olive flounder, *Paralichthys olivaceus* in Korea. J Fish Pathol.

[46] English CJ, Tyml T, Botwright NA, Barnes AC, Wynne JW, Lima PC (2019). A diversity of amoebae colonise the gills of farmed Atlantic salmon (*Salmo salar*) with amoebic gill disease (AGD). Eur J Protistol.

[47] Volkova E, Volcker E, Clauss S, Bondarenko N, Kudryavtsev A (2019). *Paramoeba aparasomata* n. sp., a symbiont-free species, and its relative *Paramoeba karteshi* n. sp. (Amoebozoa, Dactylopodida). Eur J Protistol.

[48] Karlsbakk E. Amøbisk gjellesykdom (AGD)–litt om den nye plagen. I. Bakketeig. IE, Gjøsæter, H, Hauge, M, Sunnset, BH ogToft, KØ (red) Fisken og havet, særnummer. 2015:1–2015.

[49] Kudryavtsev A, Pawlowski J, Hausmann K (2011). Description of *Paramoeba atlantica* n. sp (Amoebozoa, Dactylopodida)—a marine amoeba from the eastern Atlantic, with emendation of the dactylopodid families. Acta Protozool.

[50] Martin RE (1985). Population-growth in stationary and suspension-culture of *Paramoeba*-*Pemaquidensis* Page (Amebida, Paramoebidae). J Protozool.

[51] Wright DW, Nowak B, Oppedal F, Bridle A, Dempster T (2015). Depth distribution of the amoebic gill disease agent, *Neoparamoeba perurans*, in salmon sea-cages. Aquacult Env Interac.

[52] Preston TM, Richards H, Wotton RS (2001). Locomotion and feeding of *Acanthamoeba* at the water-air interface of ponds. Fems Microbiol Lett.

[53] Maynard BT, Taylor RS, Kube PD, Cook MT, Elliott NG (2016). Salmonid heterosis for resistance to amoebic gill disease (AGD). Aquaculture.

[54] Hytterød S, Kristoffersen AB, Darrud M, Kolstø S, Mo TA, Blindheim SH, Eide SHOG, Andersen L (2018). Standardisering av AGD-gjellescore—Enhetlig gjellescoring basert på data fra eksperimentelle forsøk og oppdrettsanlegg for laks. Vet Rapp.

