# Supplementary material for: Growth characteristics and morphology of Paramoeba perurans from Atlantic salmon Salmo salar L. and ballan wrasse Labrus bergylta in Norway
Source: Parasit Vectors. 2023 Mar 23;16:112. doi: 10.1186/s13071-023-05715-2 (PMC10037839; doi:10.1186/s13071-023-05715-2)

**Additional file 1: Alignment A1.** Alignment of the partial 18S rRNA sequences (736 bp) from the 10 clonal cultures of *P. perurans* and the sequence of *P. perurans* published by Young et al. [3] (EF216904).


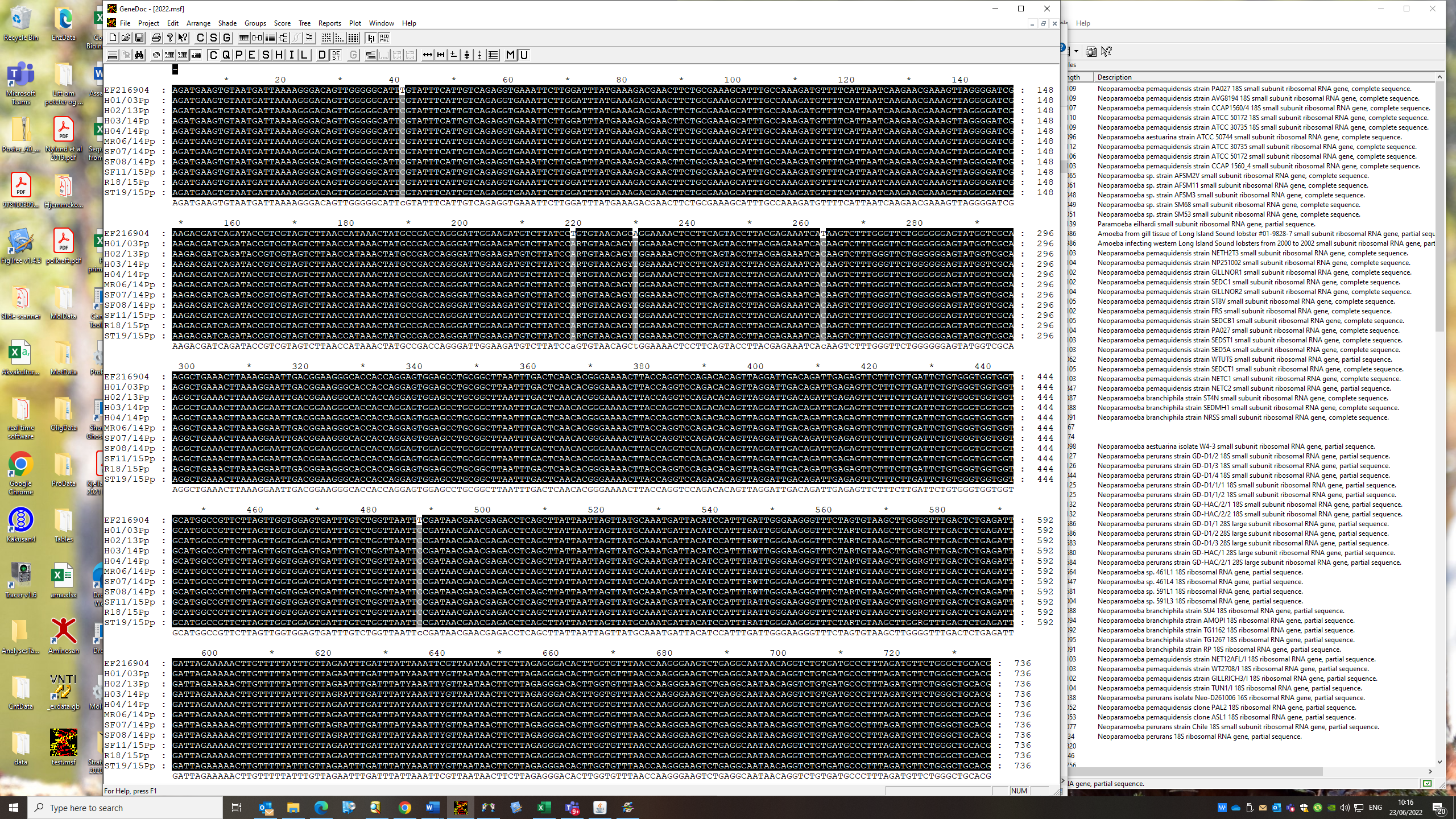

Supplement: Supplementary file 1 — Additional file 1: Alignment A1. Alignment of the partial 18S rRNA sequence (736 base pairs) from the 10 clonal cultures of Paramoeba perurans against EF216904 (from Young et al. [3]). [file 13071_2023_5715_MOESM1_ESM.docx]
